# Supplementary material for: Early Spatial and Contextual Coding Deficits in Hippocampal CA1 Precede Performance Decline in an Alzheimer's Disease Model
Source: Adv Sci (Weinh). 2026 Sep 9:e77419. Online ahead of print. doi: 10.1002/advs.77419 (PMC13558906; doi:10.1002/advs.77419)
Supplement: Supplementary file 1 [file ADVS-9999-e77419-s002.pdf]

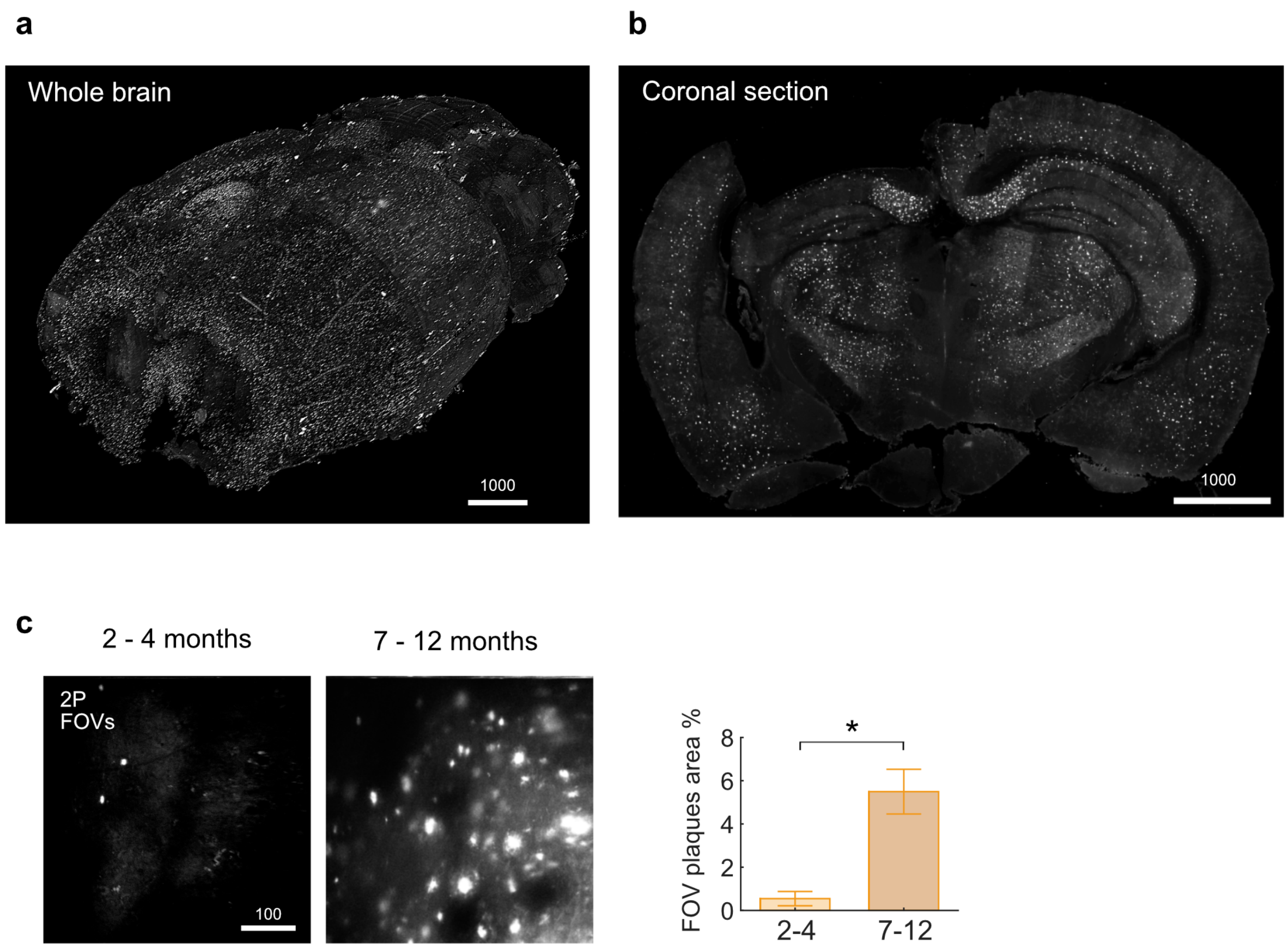

Supplementary Figure 1. Methoxy-X04 labeled Aβ plaques in 5xFAD mice

a. Three-dimensional reconstruction of a 5xFAD mouse brain labeled with Methoxy-X04, from ex vivo two-photon tomography carried out with a Tissuecyte (TissueVision, Newton MA) imaging system, complementing the MOAB-2 histochemistry shown in the main text. Scale bar, 1000 μm. **b.** Representative coronal section showing Aβ deposition in the hippocampus. The tissue opening above the dorsal hippocampus corresponds to the chronic imaging window. Scale bar, 1000 μm. **c.** Representative in vivo two-photon imaging of Methoxy-X04 labeled amyloid plaques within CA1 imaging fields of view. Quantification shows the percentage of plaque-positive area relative to the total FOV area in 5xFAD mice at median ages of 3 months (n = 3 mice) and 8 months (n = 3 mice). Data are shown as mean ± s.e.m.; comparisons were performed using a two-sided Student's t-test. Scale bar, 100 μm.

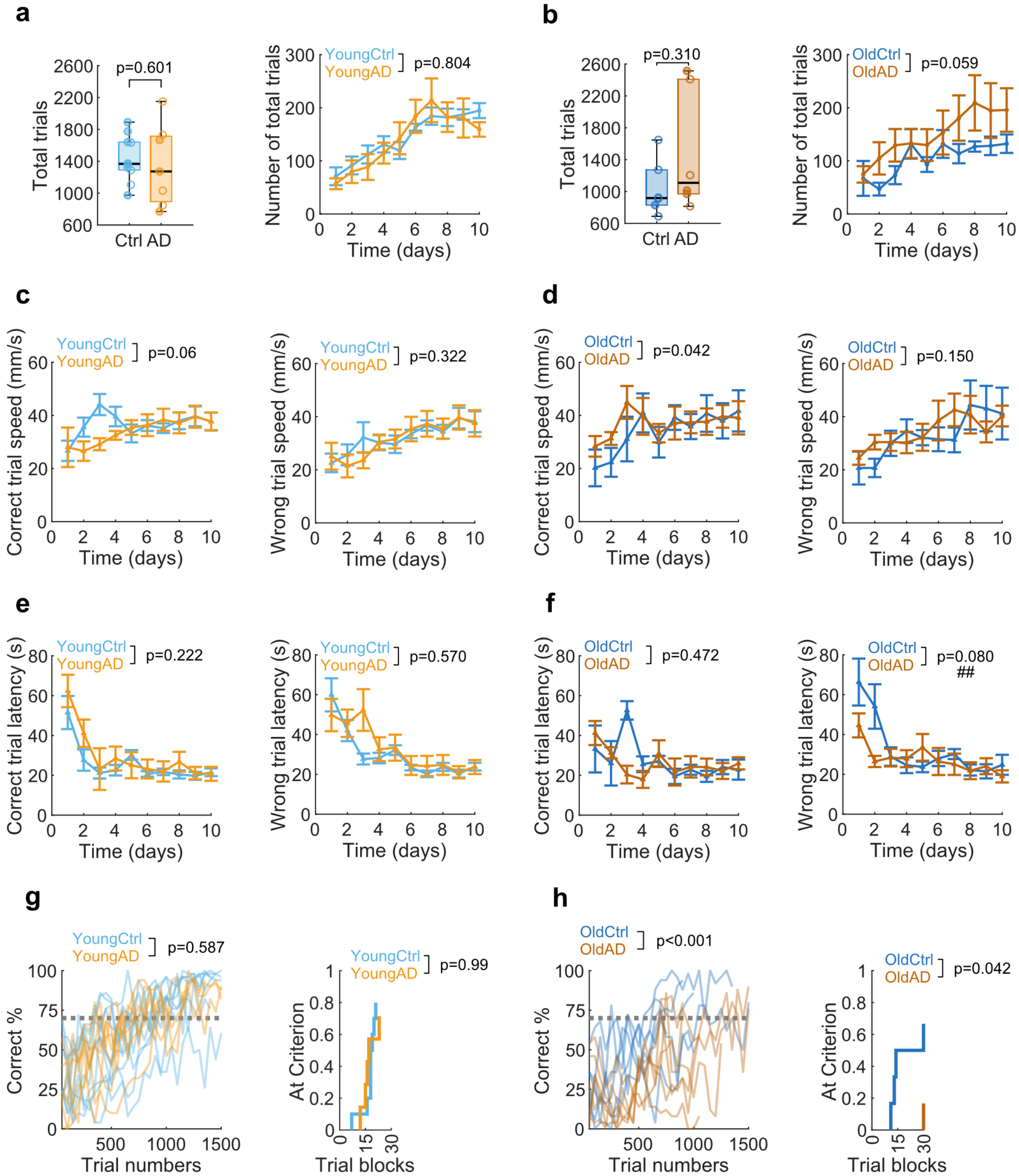

Supplementary Figure 2. Behavioral training measures in head-fixed 5xFAD mice

a. Training engagement in young control and 5xFAD mice. Left, total number of trials completed across training. Right, mean number of trials completed per training day.
b. Same as in panel a, but for old control and 5xFAD mice.
c. Running speed across training days in young mice, shown separately for correct trials (left) and error trials (right).
d. Same as in panel c, but for old mice.
e. Trial latency across training days in young mice, shown separately for correct trials (left) and error trials (right).

f. Same as in panel e, but for old mice.
g. Trial-block analysis of alternation performance in young mice. Left, individual mouse performance across consecutive 50-trial blocks. The dashed line indicates the 70% acquisition criterion. Right, cumulative proportion of mice reaching criterion across trial blocks.
h. Same as in panel **g**, but for old mice.

Data are shown as mean ± s.e.m. unless otherwise indicated. P values indicate genotype effects. Filled circles indicate male mice and open circles indicate female mice. Hash symbols indicate significant genotype × sex interactions. Statistical comparisons were performed using linear mixed effects models for training day analyses, two-sided Wilcoxon rank-sum tests for total trial counts, and log-rank tests for acquisition curves.

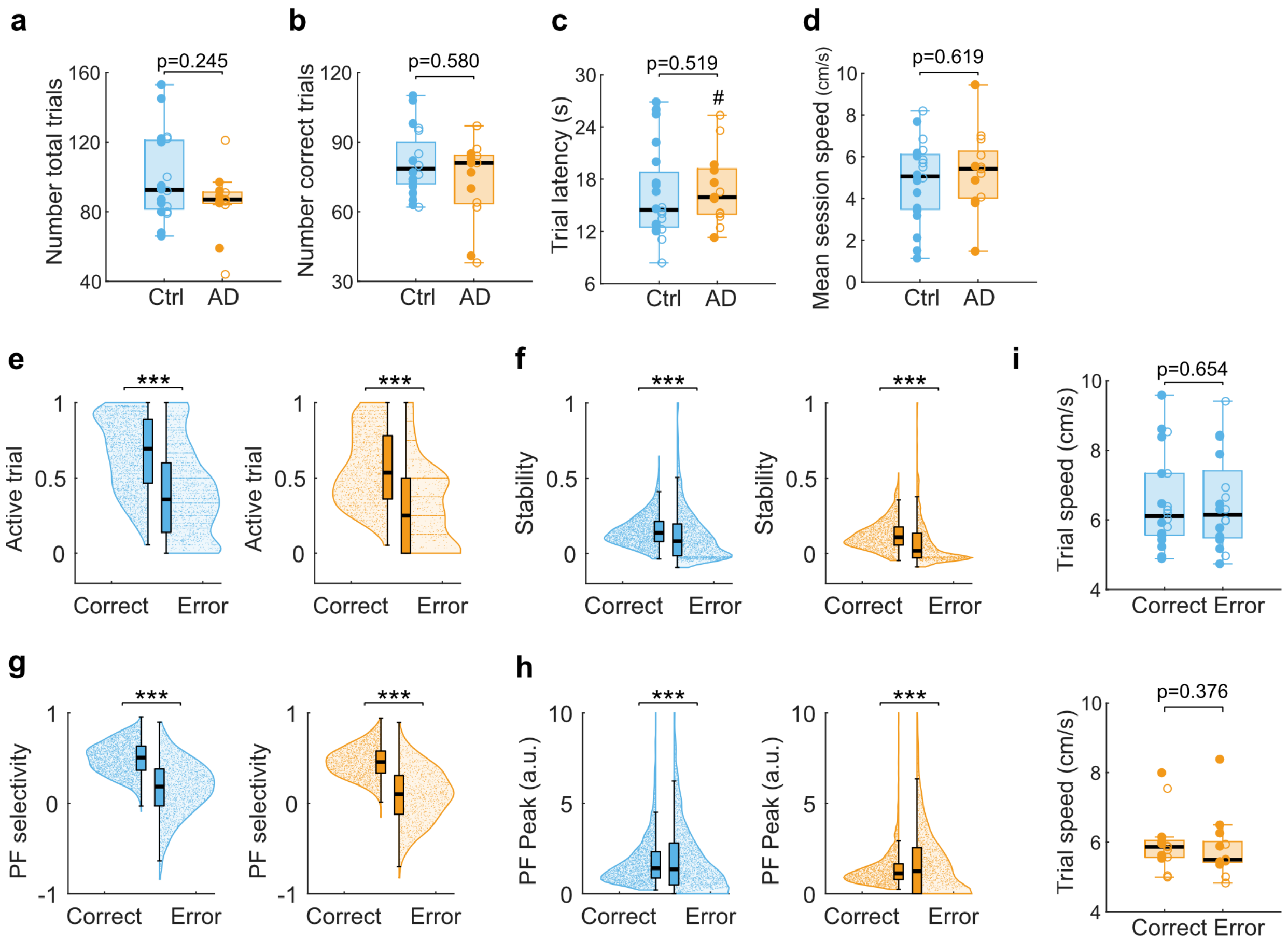

Supplementary Figure. 3 Behavioral performance and task-related place cell properties in young control and 5xFAD mice.

a-d, Behavioral measures per imaging session in young control and 5xFAD mice: (**a**) total trial number, (**b**) correct trial number, (**c**) trial latency and (**d**) mean session speed. Each point represents one imaging session.
e-h, Place cell activity properties during correct and error trials in control and 5xFAD mice: (**e**) active-trial ratio, (**f**) within-session stability, (**g**) place field selectivity and (**h**) peak in-field activity. Each point represents one cell.
i, Mean trial speed per session during correct and error trials in control and 5xFAD mice.

Control group includes 2952 cells from 20 sessions in 10 mice. 5xFAD group includes 1791 cells from 13 sessions in 7 mice. Box plots show medians and interquartile ranges, with whiskers indicating the data range. Filled circles indicate male mice and open circles indicate female mice. # indicates a genotype-by-sex interaction effect; ***P < 0.001. Statistical comparisons were performed using linear mixed-effects models for cell-level activity measures and two-sided Wilcoxon rank-sum tests for session-level behavioral measures.

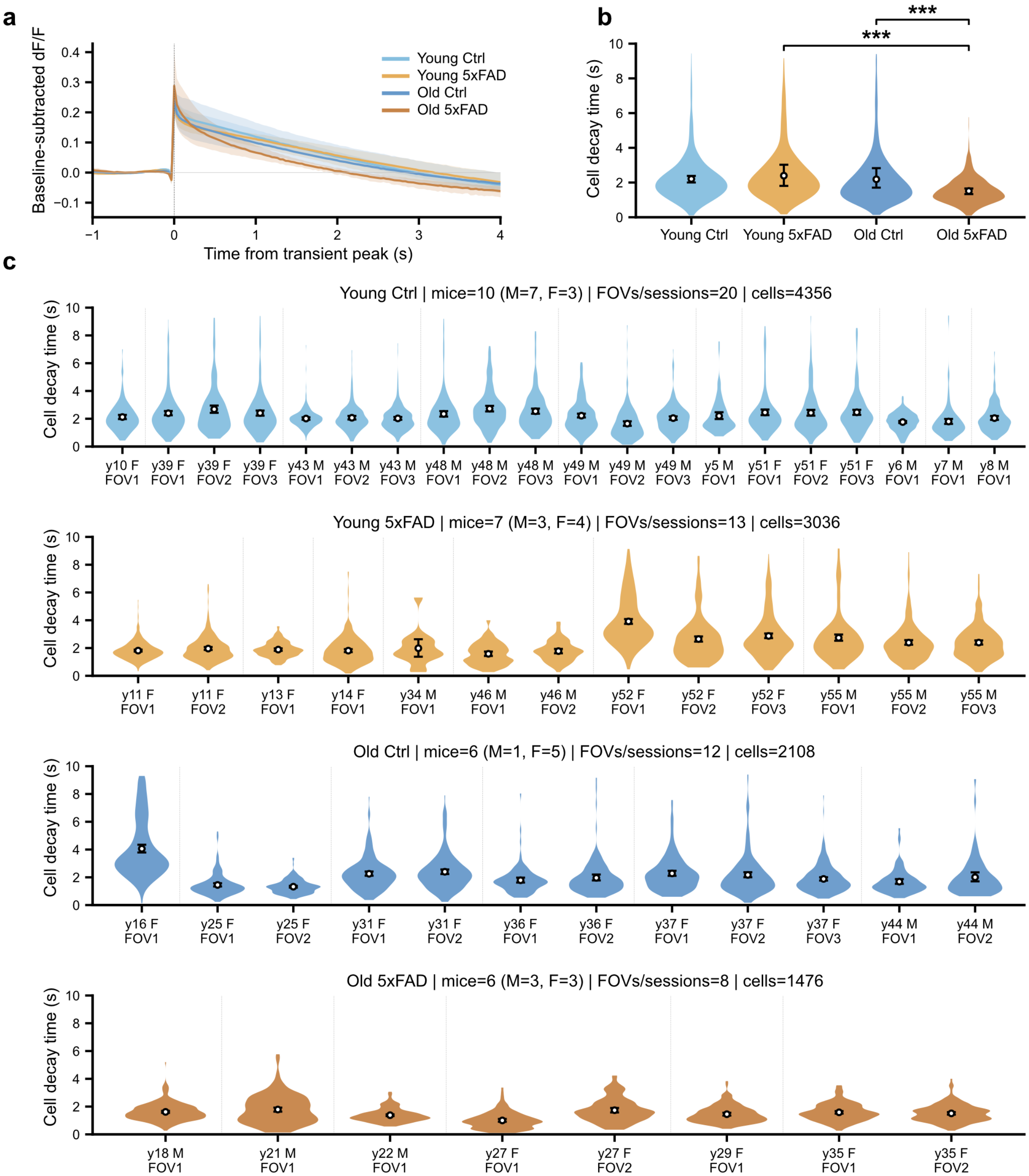

Supplementary Figure 4. Somatic calcium transient decay kinetics across age and genotype

a. Average event-triggered calcium transient decay traces across the four groups. Lines show the mean baseline-subtracted ΔF/F aligned to the transient peak, and shaded areas indicate bootstrap confidence intervals.
b. Distribution of single cell decay time across the four groups. Violin plots show pooled cell level distributions for young control, young 5xFAD, old control, and old 5xFAD mice. Open circles indicate group means, and error bars indicate bootstrap confidence intervals. ***P < 0.001.

c. Decay time distributions for individual imaging sessions. Each violin represents one FOV/session, with the corresponding mouse ID, sex, and within-mouse FOV label shown below. Dashed vertical lines separate different mice.

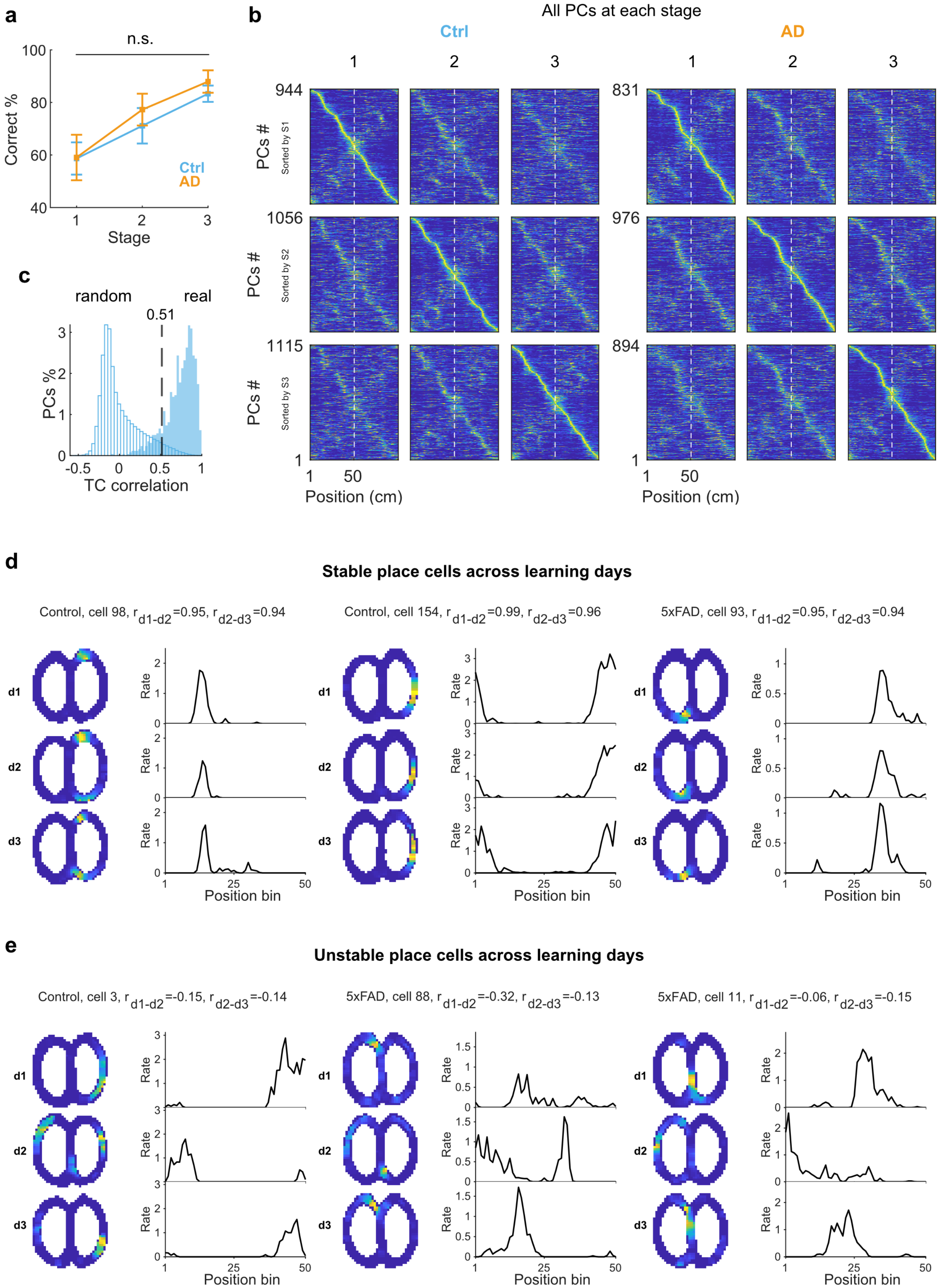
a
n.s.
Correct %
Stage
Ctrl
AD
b
All PCs at each stage
Ctrl
AD
944
1056
1115
831
976
894
PCs #
Sorted by S1
Sorted by S2
Sorted by S3
Position (cm)
c
random
real
0.51
PCs %
TC correlation
d
Stable place cells across learning days
Control, cell 98, r d1-d2 =0.95, r d2-d3 =0.94
Control, cell 154, r d1-d2 =0.99, r d2-d3 =0.96
5xFAD, cell 93, r d1-d2 =0.95, r d2-d3 =0.94
Rate
Position bin
e
Unstable place cells across learning days
Control, cell 3, r d1-d2 =-0.15, r d2-d3 =-0.14
5xFAD, cell 88, r d1-d2 =-0.32, r d2-d3 =-0.13
5xFAD, cell 11, r d1-d2 =-0.06, r d2-d3 =-0.15
Rate
Position bin

66

Supplementary Figure 5. Place cells representations across training stages

a. Behavioral alternation performance across the three imaging stages. The gray dotted line marks the 70% performance criterion.
b. Linearized spatial activity maps of place cells sorted according to peak position at stage 1, stage 2, and stage 3.
c. Tuning-curve (TC) correlation threshold used to define stable place cells across stages. The observed distribution shows within-session TC correlations between spatial tuning curves averaged across odd and even trials at stage 1. The random distribution was generated from 1,000 randomly paired cells from young control mice. The threshold for cross-stage stability was set at the 95th percentile of the random distribution (>0.51) and applied to TC correlations between adjacent imaging stages.
d. Representative stable place cells across learning stages. For each example, two-dimensional calcium activity maps and one-dimensional tuning curves are shown for stages 1-3. Adjacent-stage TC correlations are indicated above each example.
e. Same as panel d but for unstable place cells.

Data are shown as mean ± s.e.m. Control: 10 imaging sessions from 5 mice per stage. 5xFAD: 7 imaging sessions from 4 mice per stage.

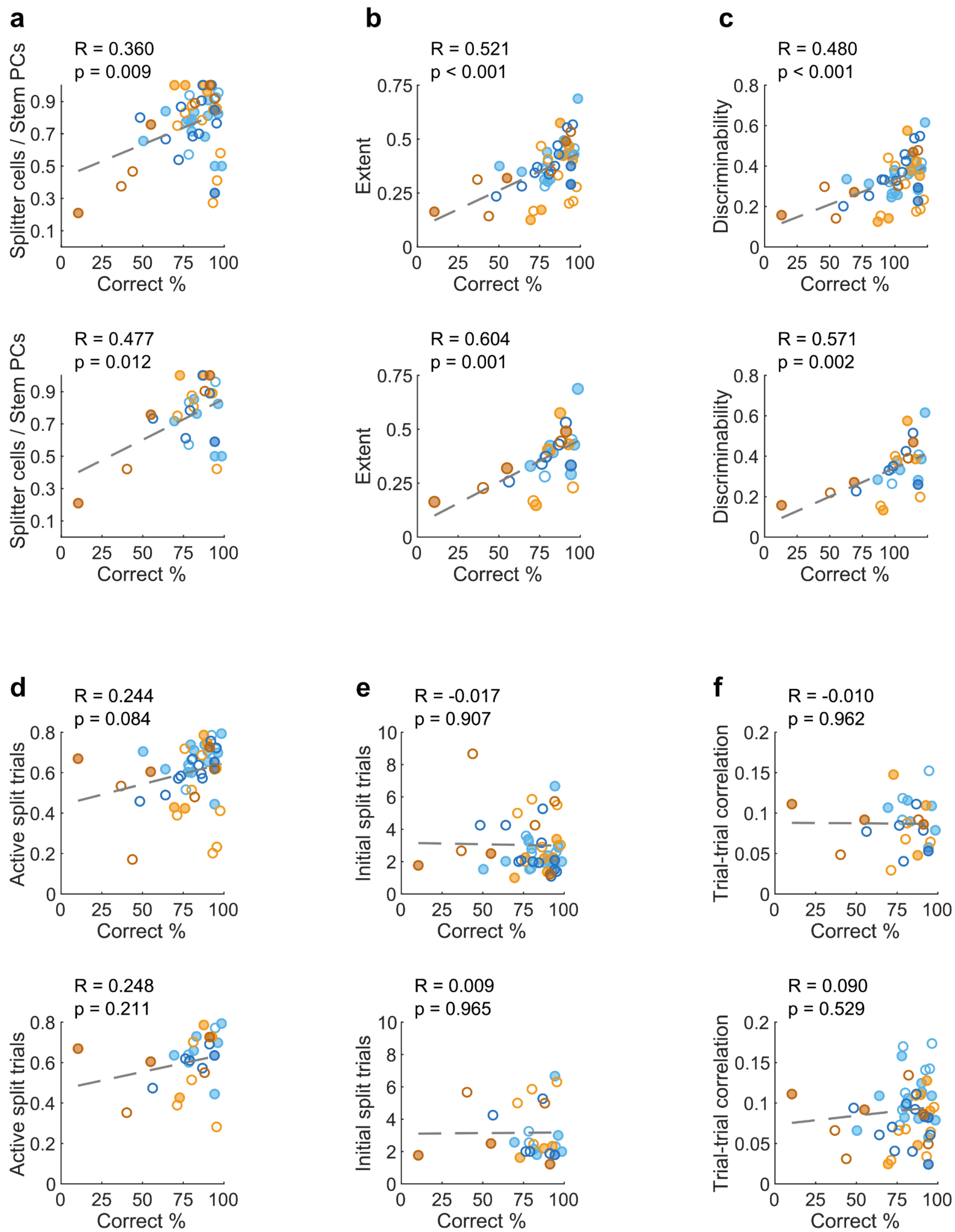

Supplementary Figure 6. Associations between splitter cell properties and behavioral performance

Pearson correlations between splitter cell metrics and behavioral performance during the alternation task. For each metric, the upper panel shows session level values and the lower panel shows mouse level values.

a. Proportion of splitter cells among stem place cells.

b. Split extent, quantified as the proportion of stem positions exhibiting significant splitting activity.

c. Split discriminability between high- and low- rate trajectories.

d. Active split trial ratio.
e. First trial exhibiting splitting activity.
f. Trial to trial spatial correlation of splitter cells.

Pearson correlation coefficients and P values are shown in each panel. Data were pooled across young control mice (20 sessions from 10 mice), young 5xFAD mice (13 sessions from 7 mice), older control mice (12 sessions from 6 mice), and older 5xFAD mice (8 sessions from 6 mice). Colors indicate age and genotype group. Filled and open circles indicate male and female mice, respectively.

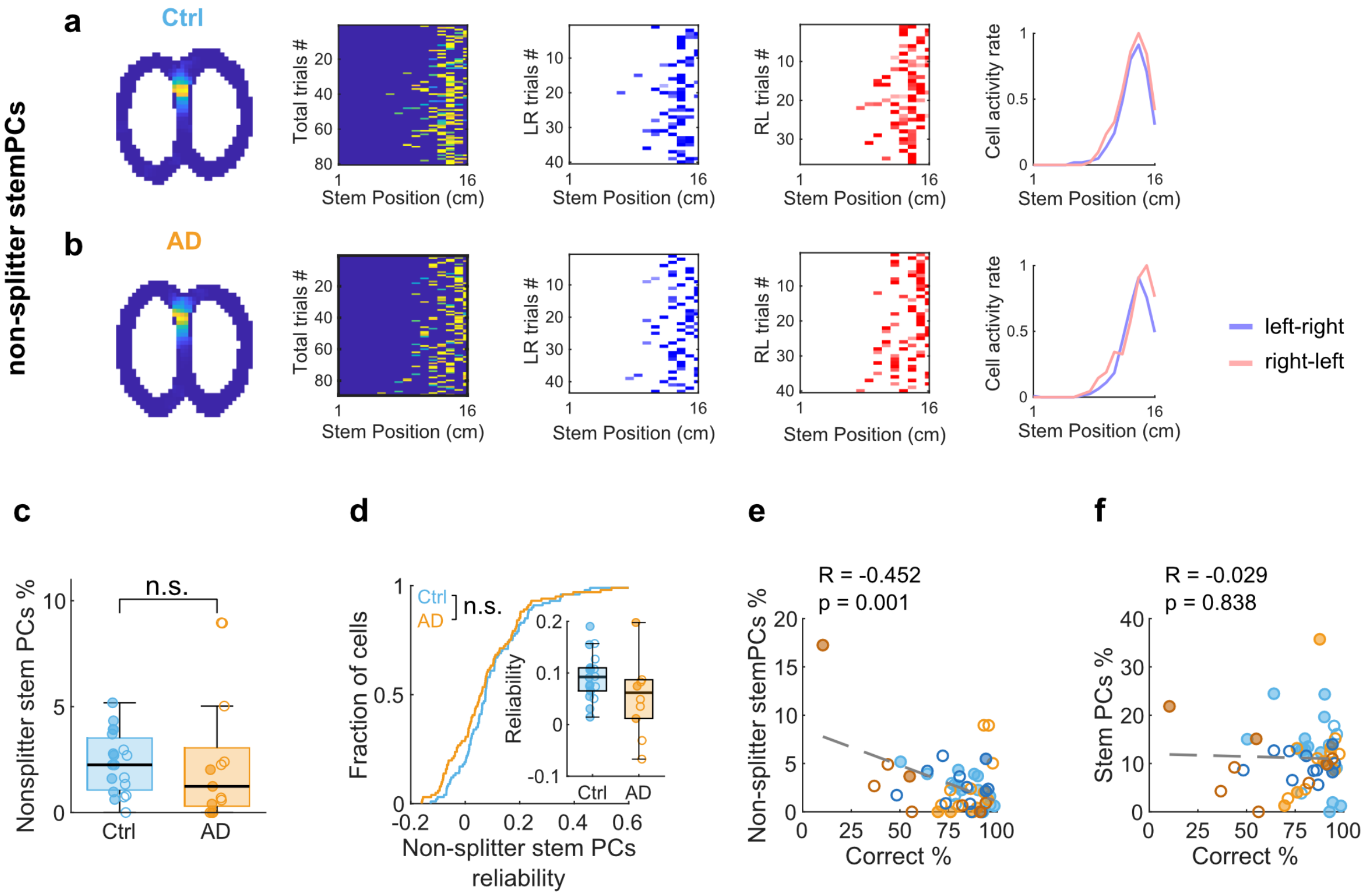

Supplementary Figure 7. Non-splitter stem place cells

a. Representative non-splitter stem place cell from a control mouse. From left to right, normalized two-dimensional calcium activity map on the Phi-maze from one imaging session, calcium activity across stem positions for each trial together with the corresponding linearized behavioral trajectory, calcium activity across stem positions for left-to-right trials, calcium activity across stem positions for right-to-left trials, and normalized calcium tuning curves for the two turn directions.
b. Same as in panel **a**, but for an age matched 5xFAD mouse.
c. Proportion of non-splitter stem place cells in control and 5xFAD mice.
d. Trial-to-trial spatial reliability of non-splitter stem place cells.
e. Correlation between the proportion of non-splitter stem place cells and behavioral performance.
f. Correlation between the proportion of all stem place cells and behavioral performance.

Data includes 53 imaging sessions from 29 mice. Non-splitter stem place cells included 146 cells from 32 sessions in 16 control mice and 177 cells from 21 sessions in 13 5xFAD mice. Blue and orange circles indicate control and 5xFAD mice, respectively. Filled and open circles indicate male and female mice, respectively.

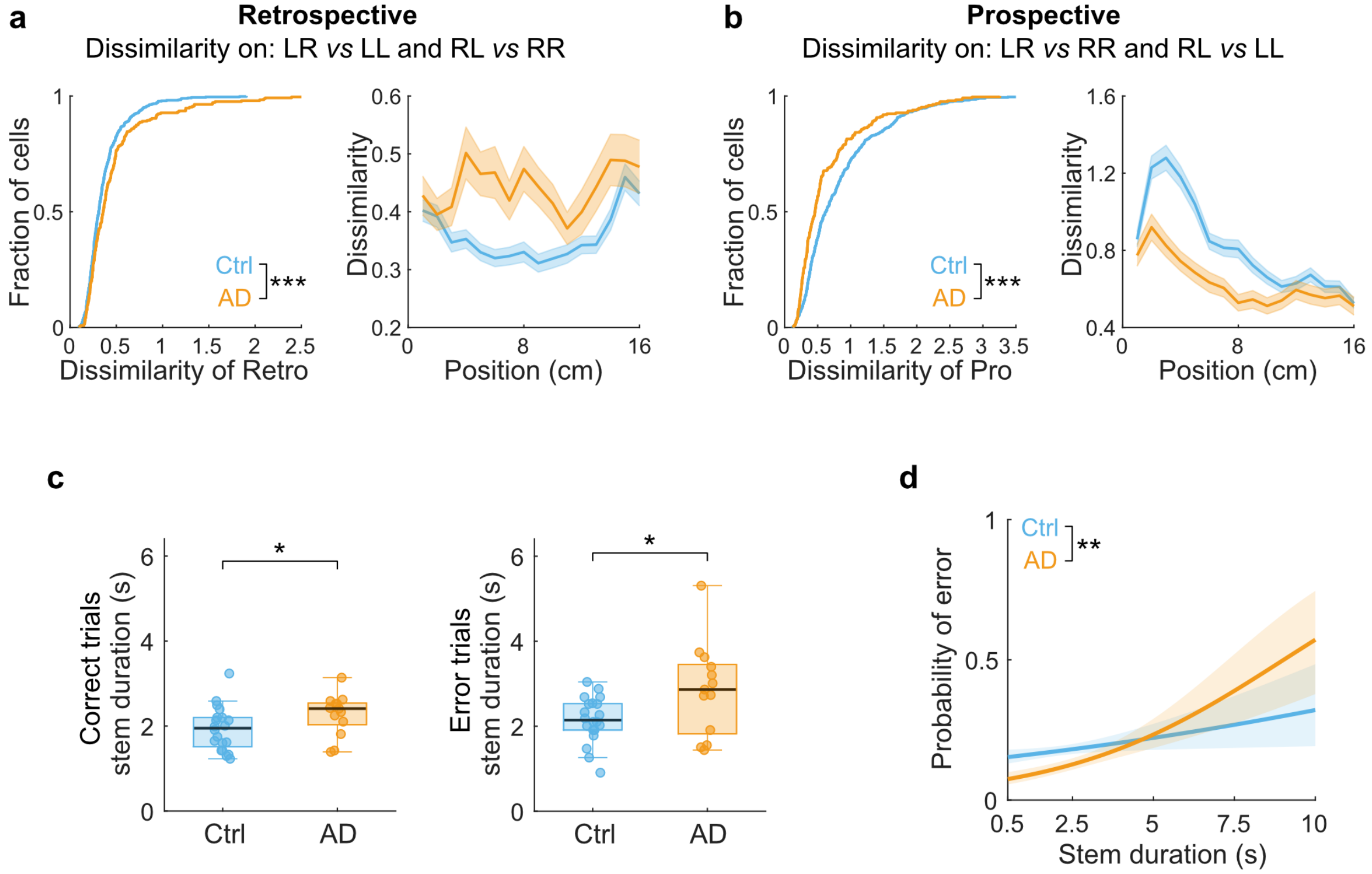

Supplementary Figure 8. Retrospective and prospective components of trajectory-dependent coding

a. Left, cumulative distribution of retrospective dissimilarity values, calculated from trial pairs sharing the same preceding trajectory (LR versus LL and RL versus RR). Dissimilarity was defined as −log10(p), where p was obtained using a Wilcoxon rank-sum test comparing cell activity rates between trial types at each spatial bin. Right, retrospective dissimilarity along the common stem. Lower values indicate greater similarity between trials sharing the same preceding trajectory and therefore stronger retrospective coding. Genotype effect, $F(1, 8981) = 40.55$, $p < 0.001$, genotype × spatial bin interaction, $F(15, 8981) = 2.08$, $p = 0.008$, two-way ANOVA.
b. Same analysis as in panel a, but for prospective coding, calculated from trial pairs sharing the same upcoming trajectory (e.g., LR versus RR and RL versus LL). Lower dissimilarity indicates greater similarity between trials sharing the same upcoming trajectory and therefore a stronger prospective component. Genotype effect, $F(1, 9064) = 144.46$, $p < 0.001$, Genotype × Spatial Bin interaction, $F(15, 9064) = 4.26$, $p < 0.001$.
c. Left, Session median stem traversal duration during correct trials in control and 5xFAD mice. Right, same as Left but for error trials. Each point represents one imaging session. Control, 20 sessions from 10 mice; 5xFAD, 13 sessions from 7 mice. Two-sided Wilcoxon rank-sum test.
d. Predicted probability of an error as a function of stem traversal duration. Solid lines and shaded bands indicate probabilities estimated using a trial-level binomial generalized linear model and their 95% confidence intervals, respectively. The model included stem duration, genotype, and their interaction (Error ~ Stem duration × Genotype) and was restricted to trials with stem durations of 0.5–10 s. The stem duration × genotype interaction was significant ($\beta = 0.194$, $p = 0.0059$). The analysis included 1945 control trials and 1076 5xFAD trials.

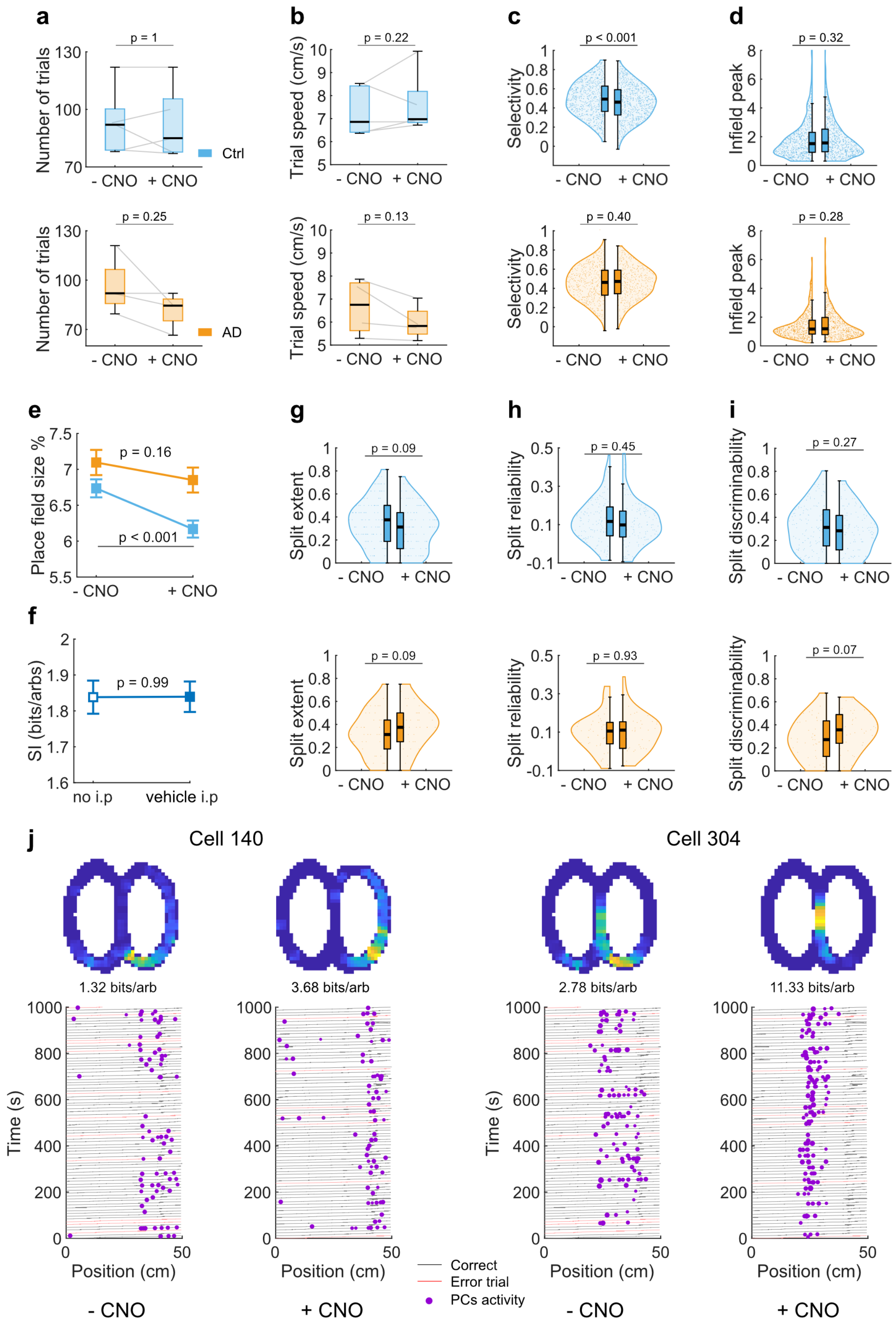

Supplementary Figure 9. Behavioral and CA1 coding measures following medial septal cholinergic activation

a. Number of trials completed following vehicle or CNO administration in control and 5xFAD mice.
b. Mean trial speed following vehicle or CNO administration.
c. Place field selectivity of CA1 place cells following vehicle or CNO administration.
d. Peak in-field activity of CA1 place cells following vehicle or CNO administration.
e. Place-field size of CA1 place cells following vehicle or CNO administration.
f. Spatial information of CA1 place cells during correct trials following no intraperitoneal (i.p.) injection or vehicle i.p. injection in hM3Dq-expressing mice.
g. Split extent of stem place cells following vehicle or CNO administration.
h. Trial-to-trial spatial reliability of splitter cells on the high-rate trajectory following vehicle or CNO administration.
i. Split discriminability between high- and low-rate trajectories following vehicle or CNO administration.
j. Representative examples of CA1 place cell activity following vehicle and CNO administration in a 5xFAD mouse. Magenta dots indicate calcium events, gray lines indicate correct trials, and red lines indicate error trials. Cell 140 exhibited a place field on the reward arm, whereas cell 304 exhibited a stem place field with trajectory-dependent splitting activity.

Data were analyzed using linear mixed-effects models with treatment (CNO versus vehicle) as a fixed effect and mouse identity as a random intercept. Ctrl, 964 detected neurons from 5 mice; 697 and 522 place cells, including 140 and 122 stem place cells, following vehicle and CNO administration, respectively. 5xFAD, 777 detected neurons from 4 mice, 522 and 449 place cells, including 46 and 41 stem place cells, following vehicle and CNO administration, respectively.

Mouse ID	Genotype	Age	Group	Gender	Session /FOV ID	Total cells	Place cells	Correct trials place cells	Splitter cells
y5	Ctrl	3.7	Young	Male	1	88	4	16	0
y6	Ctrl	3.7	Young	Male	1	309	26	87	3
y7	Ctrl	3.7	Young	Male	1	162	11	11	1
y8	Ctrl	3.7	Young	Male	1	187	73	92	14
y10	Ctrl	2.96	Young	Female	1	182	9	59	4
y39	Ctrl	3.06	Young	Female	1	334	178	206	34
y39	Ctrl	3.06	Young	Female	2	169	87	124	17
y39	Ctrl	3.06	Young	Female	3	249	142	176	30
y43	Ctrl	3.06	Young	Male	1	229	198	210	47
y43	Ctrl	3.06	Young	Male	2	222	182	200	49
y43	Ctrl	3.06	Young	Male	3	270	220	244	43
y48	Ctrl	3.6	Young	Male	1	193	81	125	19
y48	Ctrl	3.6	Young	Male	2	180	117	148	18
y48	Ctrl	3.6	Young	Male	3	231	120	161	25
y49	Ctrl	3.6	Young	Male	1	259	148	200	24
y49	Ctrl	3.6	Young	Male	2	179	97	130	15
y49	Ctrl	3.6	Young	Male	3	267	159	207	30
y51	Ctrl	3.6	Young	Female	1	225	174	195	37
y51	Ctrl	3.6	Young	Female	2	188	116	147	26
y51	Ctrl	3.6	Young	Female	3	274	171	214	42
y11	AD	4.6	Young	Female	1	334	87	106	29
y11	AD	4.6	Young	Female	2	266	102	155	29
y13	AD	4.6	Young	Female	1	145	17	33	3
y14	AD	2.5	Young	Female	1	342	96	130	14
y34	AD	3.96	Young	Male	1	14	13	14	5
y46	AD	2.5	Young	Male	1	99	20	59	4
y46	AD	2.5	Young	Male	2	78	7	27	1
y52	AD	3.6	Young	Female	1	418	120	240	29
y52	AD	3.6	Young	Female	2	268	64	184	9
y52	AD	3.6	Young	Female	3	369	85	256	23
y55	AD	2.9	Young	Male	1	247	164	203	28
y55	AD	2.9	Young	Male	2	250	174	207	25

y55	AD	2.9	Young	Male	3	243	136	177	18
y16	Ctrl	10.2	Old	Female	1	268	113	139	15
y25	Ctrl	9.07	Old	Female	1	164	55	69	13
y25	Ctrl	9.07	Old	Female	2	207	96	155	14
y31	Ctrl	8.2	Old	Female	1	229	79	139	13
y31	Ctrl	8.2	Old	Female	2	235	79	144	14
y36	Ctrl	7.6	Old	Female	1	116	34	73	8
y36	Ctrl	7.6	Old	Female	2	142	54	83	12
y37	Ctrl	7.6	Old	Female	1	169	75	124	13
y37	Ctrl	7.6	Old	Female	2	228	110	150	22
y37	Ctrl	7.6	Old	Female	3	244	97	164	19
y44	Ctrl	12.5	Old	Male	1	93	27	66	11
y44	Ctrl	12.5	Old	Male	2	73	17	38	2
y18	AD	8.37	Old	Male	1	284	106	109	13
y21	AD	7.63	Old	Male	1	182	69	109	18
y22	AD	7.63	Old	Male	1	245	80	157	28
y27	AD	9.07	Old	Female	1	151	32	51	8
y27	AD	9.07	Old	Female	2	109	29	55	11
y29	AD	8.2	Old	Female	1	156	5	16	0
y35	AD	7.6	Old	Female	1	163	17	53	7
y35	AD	7.6	Old	Female	2	187	30	103	3

Supplementary table 1. The number of identified units for each mouse and each recorded FOV/session
